# Consumers’ Perceptions of the Design of Front-of-Package Warning Labels—A Qualitative Study in China

**DOI:** 10.3390/nu15020415

**Published:** 2023-01-13

**Authors:** Xiaofan Zhang, Yifei Ouyang, Xuejun Yin, Jing Bai, Run Zhang, Jiguo Zhang, Qi Wang, Huijun Wang

**Affiliations:** 1 National Institute for Nutrition and Health, Chinese Center for Disease Control and Prevention, Key Laboratory of Trace Element Nutrition, National Health Commission of the People’s Republic of China, Beijing 100050, China; 2 The George Institute for Global Health, University of New South Wales, Sydney, NSW 2052, Australia

**Keywords:** qualitative study, warning label, focus group discussions, China

## Abstract

The warning label is identified as a simple front-of-package labeling format that assists consumers in making healthier food choices at the point of purchase. The color and/or shape of the design elements of the packaging labels can influence the understanding and purchase intention outcomes. This study aimed to explore the perceptions of consumers regarding differently designed warning labels (WLs) to identify a possibly suitable warning label design in the Chinese context. Using a qualitative approach, we collected data through 12 focus group discussions consisting of 116 participants residing in 6 provinces. Participants were selected by purposive sampling. Data were coded following inductive thematic analysis and summarized into three themes: (1) perceived preference for the appearance of the WLs, (2) perceived benefits of the WLs, and (3) suggestions for promoting the WLs. Participants preferred the black shield label showing a high content of the nutrients of concern (e.g., sodium, added sugar, and saturated fat) in both Chinese and English. Consumers generally agreed that the WLs were easy to understand and offered a simple method for choosing healthier foods. They anticipated that WLs could encourage the food industry to reformulate their products. Our study results will support the design and facilitate the use of WLs in China.

## 1. Introduction

Non-communicable diseases (NCDs) are the leading causes of death and disability globally, as well as in China [1,2]. A suboptimal diet is largely responsible for the increasing risk of NCDs [3]. According to Scientific Research Report on Chinese Dietary Guidelines (2021) [4], Chinese residents are consuming excessive sodium, fats, and added sugars. China is undergoing a “nutrition transition,” showing a shift in consumption patterns from a traditional diet based on fruits, vegetables, unprocessed grains, and legumes to a diet increasingly dominated by highly processed packaged food and beverage products [5]. Furthermore, a comparative study from 12 countries reported that packaged food in China contained higher sodium, fat, and sugar than those in other countries [6]. A recent study showed that processed foods (PFs) and ultra-processed foods (UPFs) accounted for three-fourths of pre-packaged foods in China, and the majority of PFs and UPFs exceeded the threshold for at least one critical nutrient [7]. Efforts are warranted to improve the healthiness of packaged foods in China to reduce the risk of NCDs among its population.

Consumption of unhealthy food is a modifiable risk factor. Food labeling could reduce the information asymmetry between consumers and food producers to help consumers better understand the nutritional information of food and thus make health-conscious food choices [8,9]. As a simpler, more attention-grabbing form of communicating the nutritional content and relative healthfulness of food products, front-of-package (FOP) labeling is recommended by the World Health Organization (WHO) as one of the “Best Buy” (i.e., cost-effective) interventions to combat NCDs [10,11]. FOP labels have been adopted by many countries, and these may help consumers make healthier food choices [12,13]. There are two categories of FOP labels: reductive labels (e.g., Guideline Daily Amounts (GDA)) and interpretive labels (e.g., Health Star Rating, Multiple Traffic Lights (MTL), and warning labels (WLs), etc.) [14]. As a relatively new format, WLs have been proposed that only highlight key nutrients that have exceeded recommended levels, with the aim of alerting the population about the negative health effects that these excessive nutrients may have [15]. Multiple scientific studies have shown that, compared to other FOP labels, WLs are the superior option for capturing consumers’ attention, as they are easy to understand across various populations, helping consumers identify products high in nutrients of concern, and thus discouraging them from purchasing these products. WLs are seen as an effective FOP labeling system to inform consumer decisions [15,16,17,18,19,20,21,22]. This system has been implemented across Chile, Peru, and Mexico, and passed in Uruguay and Brazil. Results from Chile showed that policies including warnings were effective in significantly reducing the purchase of sugar-sweetened beverages [23] and promoting reformulation of products to reduce sugar and sodium content [24].

Improved consumer outcomes may be attributed to the design elements of WLs [25]. The design of WLs, which consists of simple language and a widely recognized, colored alert symbol, makes them simple to recognize and understand, even among consumers with low education levels [26,27]. The WL design, including the color, shape, phrasing, and other elements, varies from country to country. For example, as the first country to implement WL, Chile’s WL is comprised of a black octagon with white borders, and the white signal phrase ‘High in/Alto en’ indicates excess nutrients. The WL designed by Brazilian researchers boasts a black triangle with white lettering and a white square background, which is thought to be more likely to attract consumers’ attention than the Chilean design [28]. Nonetheless, it is unclear which WL design would be most effective in helping Chinese consumers identify unhealthy products and discourage them from buying them. The purpose of this study is to explore the perceptions of Chinese adults regarding four different designs for WLs through a qualitative study, so as to determine a possibly suitable WL considering Chinese culture, providing a scientific basis for promoting the introduction of warning label policy in China.

## 2. Materials and Methods

We adopted a qualitative study design, using focus group discussions to collect data. The study required an in-depth understanding of consumers’ views of WLs. Focus group discussions could provide rich data, eliciting social norms, feelings, attitudes, and perceptions. The materials and methods of this study were reported following the Consolidated Criteria for Reporting Qualitative Research (COREQ) [29].

### 2.1. Study Setting and Participants

We collected data from 12 focus groups consisting of 116 participants residing in 6 provinces/municipalities (Beijing, Zhejiang, Henan, Chongqing, Guangxi, and Shaanxi) through the purposive sampling method; one urban site and one rural site were selected for each province/municipality. Interviewees were recruited according to these criteria: men and women aged 18–45 years old, with no less than two people in each age group (18–24, 25–34, 35–45), a male to female ratio of 1:1, including at least one parent, while including consumers of high-sugar and low-sugar drinks and packaged food in each group. Participants were excluded if they were health professionals, tobacco industry employees, sugary drinks and food industry workers, advertising industry workers, and those working for market research companies. Finally, 8–12 people were included in each focus group. 

All eligible participants agreed to participate in the survey, without rejection. This study was approved by the BRANY SBER IRB Ethics Committee (Approval No. IRB00010793), and all participants provided signed informed consent before the study was conducted.

### 2.2. Label Design

**Figure 1:**
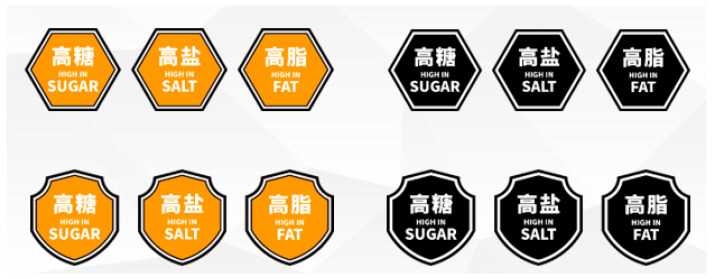
Image of four warning labels designed for Chinese consumers.

The label design was decided by the expert committee after two rounds of thorough discussion. The expert committee comprised 26 members from various professional areas, including public health, nutrition, demographics, and food policy. By comparing the shape, color, language and other elements of the label design, the committee preferred a “hexagonal” and “shield” shape, “white signal phrase on yellow or black background” color, “mixed Chinese and English” text, and agreed that “the Chinese and English fonts should be the same size for key nutrients, while the other English fonts should be smaller”. Finally, four groups of WLs were selected, which were composed of a hexagon or shield shape, with a black or yellow background (Figure 1).

### 2.3. Data Collection

The interview guide (Table S1) was developed by the expert committee and aimed at achieving an in-depth understanding of the participants’ opinions and attitudes regarding the WLs from the aspects of attention, the influence on food consumption, the warning effect for unhealthy foods, the application in Chinese culture, and the final comprehensive evaluation. Trained moderators and note-takers from the CDC or nutrition society used a semi-structured focus group discussion guide and a moderator script prepared by the research team to collect data during March 2022 and June 2022 at community health service centers or neighborhood committees, with one moderator and two note-takers for each province. The moderators were masters or PhDs in nutrition or public health, and had experience in qualitative research.

A pilot face-to-face focus group discussion with eight participants was conducted in Yuzhou City, Henan Province, on 25 February 2022 to test the feasibility of the interview guidelines and the procedures for group discussion. The wording and sequencing of the questions were adjusted after the pilot group discussion. Information from the pilot group was not included in the formal data analysis. During the formal interview, small groups of eight to twelve participants were organized. In all focus groups, the national project team provided online assistance throughout the interviews, introducing the project background and purpose at the beginning. The stimulus material (4 warning labels), uniformly provided by the project team, was projected on screens to increase the understanding of the participants of the WLs. All interviews were conducted in Mandarin Chinese. COVID-19 protocols were strictly observed, and participants were kept a safe distance from each other and wore masks all the times.

Each focus group discussion was recorded with an audio-video recorder. The final transcript was created by checking the field notes and audio recordings. The preliminary analysis was iterative, concurrent with data collection, to refine the probing and assess the saturation. Saturation was achieved in the 12th focus group. 

### 2.4. Data Analysis

Data were analyzed following inductive thematic analysis by two researchers (X.Z., R.Z.); codes and themes emerged from participants’ responses [30]. The researchers read the transcripts repeatedly to familiarize themselves with the data, and they coded and analyzed the data using Nvivo (version 11.0) [31], respectively. Throughout the process, the coders met to review the coding and reached a consensus on code names and meanings. They also used the memo function in the software to record any new ideas. Any discrepancies were discussed until the final themes and codes were formed, and the two researchers also consulted with a senior author (X.Y.), if necessary. We used typical quotations from participants to support the themes presented in the results, without including any personal information.

## 3. Results

There were 12 focus group completed, with a total of 116 participants. These individuals were made up equally of males and females, with half in urban and half in rural areas. The interviewees aged 25–34 accounted for the largest proportion (n = 46, 44.2%); college or university graduates (n = 77) accounted for 66.4% of the total. There were also 62 (53.4%) parents or primary caregivers of children under the age of 16. Each group interview lasted about 2–2.5 h. From the data, we extracted three themes, with several subthemes (Table 1). Through the perception of design for WLs, consumers expressed their understanding of the effectiveness of the WLs, and additionally, put forward their own suggestions regarding the promotion of WLs.

### 3.1. Perceived Preference for the Appearance of the Warning Label

Interviewees generally believed that the text of the WLs was eye-catching and easy to understand. The black WL, designed with white phrases on a black background, was more comparable and attractive on a colorful food package. Black also reminded them of danger, such as those exhibited by chemicals and pesticides, and was effective in warning of unhealthy foods. The shield shape gave them a feeling of protection. It was viewed as more authoritative and acceptable by the consumers, compared to the shape of hexagon. From the perceptions of appearance, more consumers preferred the black shield design for the WL.

**Color.** Participants felt that black drew more attention and effectively contrasted with colorful product packages: “The color of black is more attractive to me, because the color of food package is generally gorgeous and colorful. If the yellow label is used, it will not attract people’s attention at first sight, but black is easier to see” (Guangxi, 0207). Some consumers added that the black label stands out because of its high contrast with the white text in the label: “The color of the text in the label contrasts with its black back-ground, which may make it easier to notice” (Zhejiang, 0208). Others raised the interesting point that black looks serious, dull, and scary, implying prohibition, making people less inclined to buy: “Because black first gives people a more serious and dull feeling. If you see this color, you may give priority to not choose to buy products with this label” (Chongqing, 0101). 

Black also reminded them of hazardous chemicals and pesticides: “Because black reminds me of the toxic logo on pesticide packaging, I feel like I won’t buy it” (Chongqing, 0108). More participants thought black was the most effective for warning of unhealthy food: “The darker the color, the less healthy it is, which means black is the least healthy, so for me it’s definitely black” (Zhejiang, 0108). In traditional Chinese culture, black often represented bad luck and taboo: “In fact, I think the combination of black and white, especially the large range of white text on black background, may be associated with disease and death in our traditional culture, but it is appropriate to use on warning labels” (Shaanxi, 0110). Others thought black was not appropriate in China: “I don’t think black is suitable, Chinese people may not like black” (Chongqing, 0102). “Black and white is a bit unlucky for our culture, so I didn’t choose black” (Zhejiang, 0207). For the above reasons, although some people thought black may contradict Chinese culture, the vast majority of participants thought that black was more suitable for the warning label.

A few people preferred yellow. Some participants believed yellow was a bright color and more eye-catching: “Its color is fluorescent, the contrast on the packaging is relatively bright, so it is more eye-catching” (Guangxi, 0205). Some participants thought that yellow is soft and warm, more acceptable to children, more suitable for food: “From the perspective of food, the label is just designed to remind consumers which food is high in salt, high in sugar or high in fat when they make a choice, so, label should use warmer color. What’s more, yellow on snacks is more acceptable to children” (Zhejiang, 0207). In addition, yellow reminded them of traffic lights: “In our daily life, yellow is like a yellow light at a traffic light. It tells us to wait, serves as a warning label” (Chongqing, 0208). Yellow could also remind them of the yellow code (a COVID-19-related code, wherein people with such a code may be at risk), a yellow rainstorm warning, and the yellow card warning in sports: “I think yellow is warning, because we use yellow cards a lot at sporting events” (Henan, 0101). Another participant added: “yellow reminded him of sub-health status and foods high in calories, like fried foods” (Chongqing, 0103). But others did not think yellow was appropriate for a food label: “Because in ancient China, yellow represents the color of emperors, which is not suitable for ordinary people” (Zhejiang, 0203).

**Shape.** The shape of the shield implied resistance and gave participants a sense of protection. Therefore, most consumers chose the shield shape: “In ancient times, shields were used for protection in battle” (Henan, 0109). Participants also stated that the shield represented authority and was more formal: “When you see the shape, you feel that it is authoritative, just like the authoritative test report, unlike the messy evaluation, which has a certain credibility” (Shaanxi, 0103). Participants preferred shield shape because it was less common, visually larger, and more attractive than hexagons: “The Shield is more irregular and noticeable than the hexagon, visually larger” (Zhejiang, 0109).

Some participants preferred the hexagon to be used for warning label: “I personally prefer hexagon, because shield really has a kind of protective meaning, which may let you feel that food is safe and healthy, easy to cause misunderstanding” (Shaanxi, 0110). Participants were drawn to the hexagon because of its sharp edges: “The curves are softer, the hexagon has edges and corners, just like sting me, so it stands out” (Shaanxi, 0103). Besides, the hexagon shape reminded people of the structure of sugar and dangerous chemicals. “The hexagon reminds us to eat a balanced diet, as it is a well-balanced shape”, said one participant from Zhejiang (Zhejiang, 0110). Another participant agreed with the hexagonal label: “The number six in China is better in feng shui, representing everything goes smoothly” (Zhejiang, 0205).

**Text.** Participants generally agreed that the text on the label was easy to understand and might affect their food purchases: “I think the warning label is very suitable in our country. I often see nutrition composition table when buying food. However, a lot of people probably don’t understand. If you have the warning label on it, with high in sugar, high in salt, high in fat, it will be very simple and clear. The meaning can be understood all of a sudden, which is very good” (Henan, 0209). In addition, the text on the label would warn that the food was unhealthy, and they may not buy it: “Text affects your decision because it says high in sugar, high in salt and high in fat. If you have high blood pressure, you see a label that says high in salt, you will immediately stop buying it” (Guangxi, 0203).

### 3.2. Perceived Benefits of the Warning Label

The majority of participants had a positive view of WLs, believing that WLs were easy to understand. It could help people make healthier food choices, educate people to care about their health, and encourage the food industry to reformulate their products. A few individuals felt that the effect of WLs varied from person to person. It was viewed as especially useful for high-risk populations, while participants felt that the general population, especially younger children, will consume packaged food regardless of the WL.

**Influence on consumers’ food purchases and health awareness.** The effects of WLs on food choices were bifurcated. The majority of people thought that WLs could help them choose healthier food by identifying the foods high in sugar, fat, or salt: “I think it makes sense that you can use these labels to quickly determine what type of food it is, and food labeled is definitely not good, you don’t have to look at the ingredient list” (Beijing, 0212). A participant from an urban area in Henan province said, “I think the labels can help people avoid unhealthy food when they buy food, especially for people with diseases, the labels can help them more effectively select what they need and what they don’t need” (Henan, 0101). However, some people thought that the label was of little meaning, if they really wanted to eat this food, the label would not affect their food purchases: “I don’t think it makes much sense. It doesn’t use something rotten to make you feel sick like a smoking sign in foreign country. These WLs occupy a very small area on the package, and the color do not cause much disgust” (Beijing, 0203). Another nearby participant added: “I don’t think the label means much. If people want to eat it, he will eat it. If he doesn’t want to eat it, he won’t” (Beijing, 0204).

**Warning labels also increased consumer health awareness.** WLs could encourage consumers to take care of their health: “I think it is necessary to put these labels on the front of food package. Because only after you know the content of sugar, salt and fat in food, can you pay more attention to your own health, especially now that there are more and more obese people, they are more concerned about the intake of these nutrients” (Guangxi, 0105). WLs could also remind consumers to improve their dietary habits: “This label can lower the cognitive threshold of consumers, and people can intuitively find that the things they usually eat are harmful, so as to guide them to think deeply, reflect on and change their eating habits. Therefore, I think it is suitable for promotion, and the earlier the better” (Shaanxi, 0110).

**Positive effects on food industry.** WLs could promote the food industry to reformulate their products to be healthier: “WLs is actually a boost for the food industry to produce high-quality, healthy, green food that is good for people” (Shaanxi,0103). WLs could also promote China’s food development and bring it in line with international standards: “I think this is necessary, although our country is a developing country, it is developing very fast, and it is a big country in food and agriculture. We must keep pace with other developed countries in food import and export. Many countries have implemented this label, we also need to make the development of food as soon as possible to catch up with the speed of national development, only then can our food catch up with the world, our people’s physical quality is gradually improved” (Shaanxi,0106).

**Positive effects on children’s education.** Parents could use these WLs to educate their children about which foods to eat less of, further helping children to develop healthy eating habits: “Because many children need their parents to buy food for them, when parents see these labels, they can tell their children that these foods are not healthy and ask them to eat as less as possible” (Henan, 0108). A city resident in Shaanxi mentioned that “if food could be labeled with warning labels, people may pay attention, especially children, many of the lover of snacks are children. If children can develop a good habit of not eating unhealthy snacks when they are young, they may not be interested in them when they grow up” (Shaanxi, 0108).

### 3.3. Suggestions on the Promotion of Warning Label

Finally, our participants made some suggestions for the future application of WLs in China, including label design and additional uniform specifications. They also strongly recommend greater publicity for the WL.

**Suggestions regarding label design.** It was recommended that the label should be triangular: “Personally, I think the triangle is more suitable. Hexagon is symmetrical, which gives people a sense of balanced nutrition, shield gives a sense of protection, I don’t think these shapes are suitable for a warning label. In my daily life, I come across a lot of warning signs with triangles” (Zhejiang, 0111). A variety of colors were recommended: “In my personal opinion, I recommend using different colors for different nutrients, such as orange for high sugar, red for high fat, and black for high salt”. (Guangxi, 0206). Another participant from Zhejiang province said: “The colors should be colorful, label with a brighter color to highlight national culture”. As for the text description of the label, some participants gave corresponding suggestions: “Maybe adults know that high salt, sugar and fat are harmful, but teenagers don’t. So I think if we want to introduce warning labels, we should at least add a description of the health risks of high sugar, high fat and high salt to the label, just like the text description of smoking” (Zhejiang, 0110). It was also recommended that: “The label should indicate the percentage of sugar, fat and salt in the food above the recommended value, such as 30% or 80%; Or further rank the levels of high sugar, fat and salt indicated by the depth of the color” (Zhejiang, 0208). As for the design of warning labels, participants also suggested adding food icons: “I think the English text can be changed to the icon. For example, you can use a picture of sugar instead of high sugar and a picture of meat instead of high fat, if fat people see the meat icon, they will not want to eat these products” (Zhejiang, 0105). 

**Additional uniform specifications for the warning label.** Many respondents mentioned that there should be uniform regulations on where labels should appear on food packaging: “Although they were on the front of the package, they were more visible one-third of the way up” (Shaanxi, 0103). Another participant added that: “I feel like if you put the label in the upper right or lower right of the food package, I might not notice it, but if you put it in the upper left, it really stands out” (Shaanxi, 0106). In addition to the location, the size of the WLs should also be uniform: “The size of the label must be regulated, according to the size of the package to specify the size of the label. It doesn’t have to be the same size, but it should be the right size in proportion to the package” (Guangxi, 0105). Respondents from rural Shaanxi Province also expressed the same opinion.

**Strengthen publicity on the health hazards of high sugar, salt, and fat.** Some interviewees suggested that more publicity should be promoted: “I think we need to make great efforts to popularize health knowledge, because there are still some people who do not know the meaning of high sugar, salt and fat” (Guangxi, 0104). “Publicity is also very important. Some people may be indifferent to the warning labels, because they will not feel uncomfortable immediately even if they eat the food with WLs. For example, many people have high blood pressure but don’t feel any discomfort, so they don’t care it, even don’t pay for medicine” (Shaanxi, 0103).

## 4. Discussion

This study is the first qualitative study to explore WLs suitable for China and the preferences of Chinese consumers. It was found that Chinese consumers tend to prefer a black shield warning label with words warning that the product is high in sugar, salt, or fat. The use of shapes [20] and colors [32,33] associated with danger increases the effectiveness of warning labels. In this study, Chinese consumers perceived the black shield in the warning label as depicting danger. However, in other countries, such as Chile [34,35] and Israel, consumers perceived the octagon as best communicating a warning. Consumers in Brazil [28] related triangles with danger. In this country, the octagon or triangle warning labels were better understood than those of other shapes. The association between shape and danger is interpreted differently in different cultures [36,37], indicating the need for each country to design warning labels according to its own cultural characteristics in order to improve the efficacy of the warning label.

Black is associated with hazardous chemicals, toxic pesticides, and horror films, showing an increased risk perception in participants. This color seemed to make many consumers think about the negative health effects, also triggering their feelings of fear. Previous studies have reported similar results [38,39]. According to the health belief model, labels that increase risk perception are more effective in altering people’s attitudes, which may ultimately lead to changes in their behavior [40]. Participants tended to choose black because of its contrast with the colorful product backgrounds, which is similarly reported by Cabrera et al. [37]. Visibility against the background of the product is quite important for WL effectiveness [41]. The findings of this study also confirm that consumers preferred the white words on the black background over the label with a yellow background. This is similar to the label design in Chile [35] and Uruguay [42].

In our study, participants felt that the text of the label was critical, and it should clearly state that the food is unhealthy. A recent cross-sectional study conducted to investigate the preferences of Chinese parents for different FOP labels also showed that parents thought the nutrients most in need of labeling should be sugar, salt, and total fat [43]. These three nutrients are also targeted for reduction in the “Healthy China 2030” initiative, with the ambitious goals of cutting dietary oil by 30–40%, salt by some 50%, and sugar by at least 17% nationwide by 2030 [44]. Currently, prepackaged foods in China must be labeled with salt and fat. Sugar is a mandatory new nutrient under the revised nutrition labeling standard GB28050. WHO guidance suggests that the warning labels be focused on nutrients already required to be declared on the back of the package [45]. Hence, an FOP label regarding the nutrients of salt, sugar, and fat is reasonable in China. 

This study found that Chinese consumers had a positive attitude toward WLs on prepackaged food. A warning label that is easy to see and understand will attract consumer attention [41], increase their use of the label, and produce behavioral changes [40,41,46]. Studies in Uruguay and Chile, where WLs have been implemented, showed that participants accepted WLs and found them easy to interpret [34,47]. The results of multiple quantitative studies have also found that consumers understand WLs better than other forms of labeling [48,49,50]. The WL simplifies nutritional information by directly listing nutrients occurring in high amounts. Some participants in this study felt that WLs enabled them to quickly identify products with high amounts of risk nutrients, which is supported by other experimental studies [27,51].

Regarding the perceived effect of the WLs, although some participants felt that they would continue to buy food even if it is labeled with a WL, others felt that a WL would help consumers identify unhealthy products when shopping, thus influencing their behavior. Those who felt that they would not be deterred by the WL fit the health belief model, which assumes that low risk perception does not cause behavior change. Familiarity with products also decreases the effectiveness of the warning labels [52,53]. One of the goals set by WL is to discourage purchasing unhealthy products [52], which is consistent with consumer perceptions of WLs in the current study, indicating that a WL may achieve this goal among Chinese consumers. Many studies have reported perceived changes in purchasing behavior post exposure to WLs. An online experimental study in the UK showed that image-based warning labels discourage sugar sweetened beverages selection by parents [54]. Another similar study in Colombia reported the warning label most deterred participants from purchasing products “high in” risky nutrients compared to other front-of-package labels [55]. In addition to the online simulation, real-world quantitative studies from Chile also revealed an 8.0% and 1.2% decrease in chocolate and cookie sales following the implementation of WLs, further confirming the effect of WLs on unhealthy purchasing behavior [56]. Reducing unhealthy food purchases will help lower the prevalence of non-communicable diseases and obesity.

Participants in the current study agreed on the importance of making healthier food choices for children, such as helping children develop healthy eating habits at an early age, a positive step in the direction of behavior change [20]. As cardiovascular diseases occur at a younger age [57], parents need to make smart food choices in order to inculcate healthy dietary patterns in their children at an earlier age. Our participants agreed that simple warning labels would also help children make healthy choices independently. A study in Uruguay reported that the impact of WLs on children’s food choices was higher than the impact reporting using the traffic-light system [58]. A qualitative study in Brazil found that WLs had the potential to support healthier behaviors in both consumers and their children [15]. This observation is important, because reducing childhood obesity by reducing the consumption of unhealthy packaged foods is urgent.

The strength of this study is that the data were collected in six provinces, including urban and rural areas, taking into account the views of consumers from diverse sociodemographic backgrounds. Moreover, this is the first qualitative study to explore warning labels suitable for the Chinese culture regarding consumer perspectives; participants in the study also offered many good suggestions, providing data support for the implementation of WLs in China. The limitation of this study is that focus group discussions can be easily influenced by one of the panelists, which is common in qualitative research. Besides, this study was experimental, and the actual effect can only be determined once the WLs are implemented in China. More studies are needed in the future to clarify the hindering and promoting factors of the implementation of WLs and the influence of WLs on actual purchasing behavior.

## 5. Conclusions

In conclusion, our results from focus groups in China suggest that a policy placing warning labels on the front of unhealthy packaged foods could improve consumers’ understanding of health risks and help them identify unhealthy foods; it could also promote children’s diet education and the development of the food industry. Certain design elements, such as color (black), shape (shield), and high contrast with the white text could enhance a label’s ability to increase the perception of risk. Suggestions regarding the promotion of WLs were also made by the participants, such as standardizing the location and size of the label, indicating more detailed information about the nutrients concerned, and suggestions regarding the color, shape, icons, and text description of the label. Chinese policymakers might consider the black shield warning as part of a front-of-package labeling policy. More research on the impact of WL implementation is needed in the future.

## Figures and Tables

**Table 1:** Themes and subthemes.

Themes	Subthemes
Perceived preference for the appearance of WLs	Color
	Shape
	Text
Perceived benefits of WLs	Influence on consumers’ food purchases and health awareness
	Positive effects on food industry
	Positive effects on children’s education
Suggestions for promoting WLs	Suggestions for label design
	Additional uniform specifications for warning label
	Strengthen publicity on the health hazards of high sugar, salt, and fat content

## Data Availability

Data will be made available with the consent of the corresponding author.

## Supplementary Materials

The following supporting information can be downloaded at: https://www.mdpi.com/article/10.3390/nu15020415/s1, Table S1: Six core questions in the interview guide.
