# Supplementary material for: Consumers’ Perceptions of the Design of Front-of-Package Warning Labels—A Qualitative Study in China
Source: Nutrients. 2023 Jan 13;15(2):415. doi: 10.3390/nu15020415 (PMC9866720; doi:10.3390/nu15020415)
Supplement: Supplementary file 1 [file nutrients-15-00415-s001.zip › nutrients-2095852-supplementary.pdf]

Table S1. Six core questions in the interview guide

Number	Content
1	Which group of warning label did you choose regarding "most attracted your attention"? Why is that? What caught your attention?
2	Which group did you choose regarding the "labels most likely to influence your decision to consume food or beverage"? Why is that? What factors of label influenced your decision?
3	Which group do you think we just showed is the most effective for warning people about unhealthy food, and why? Why do you think it works?
4	Do you think these labels are suitable for use in our country?
5	Are there any labels that are incompatible with our culture or inappropriate for use in our country?
6	Evaluate all labels comprehensively
